# Ubiquinone Binding and Reduction by Complex I—Open Questions and Mechanistic Implications

**DOI:** 10.3389/fchem.2021.672851

**Published:** 2021-04-30

**Authors:** Etienne Galemou Yoga, Jonathan Schiller, Volker Zickermann

**Affiliations:** ^1^Institute of Biochemistry II, University Hospital, Goethe University, Frankfurt, Germany; ^2^Centre for Biomolecular Magnetic Resonance, Institute for Biophysical Chemistry, Goethe University, Frankfurt, Germany

**Keywords:** respiratory chain, NADH dehydrogenase, oxidative phosphorylation, proton pumping, electron transfer, semiquinone, inhibitor

## Abstract

NADH: ubiquinone oxidoreductase (complex I) is the first enzyme complex of the respiratory chain. Complex I is a redox-driven proton pump that contributes to the proton motive force that drives ATP synthase. The structure of complex I has been analyzed by x-ray crystallography and electron cryo-microscopy and is now well-described. The ubiquinone (Q) reduction site of complex I is buried in the peripheral arm and a tunnel-like structure is thought to provide access for the hydrophobic substrate from the membrane. Several intermediate binding positions for Q in the tunnel were identified in molecular simulations. Structural data showed the binding of native Q molecules and short chain analogs and inhibitors in the access pathway and in the Q reduction site, respectively. We here review the current knowledge on the interaction of complex I with Q and discuss recent hypothetical models for the coupling mechanism.

## Introduction

**Figure 1:**
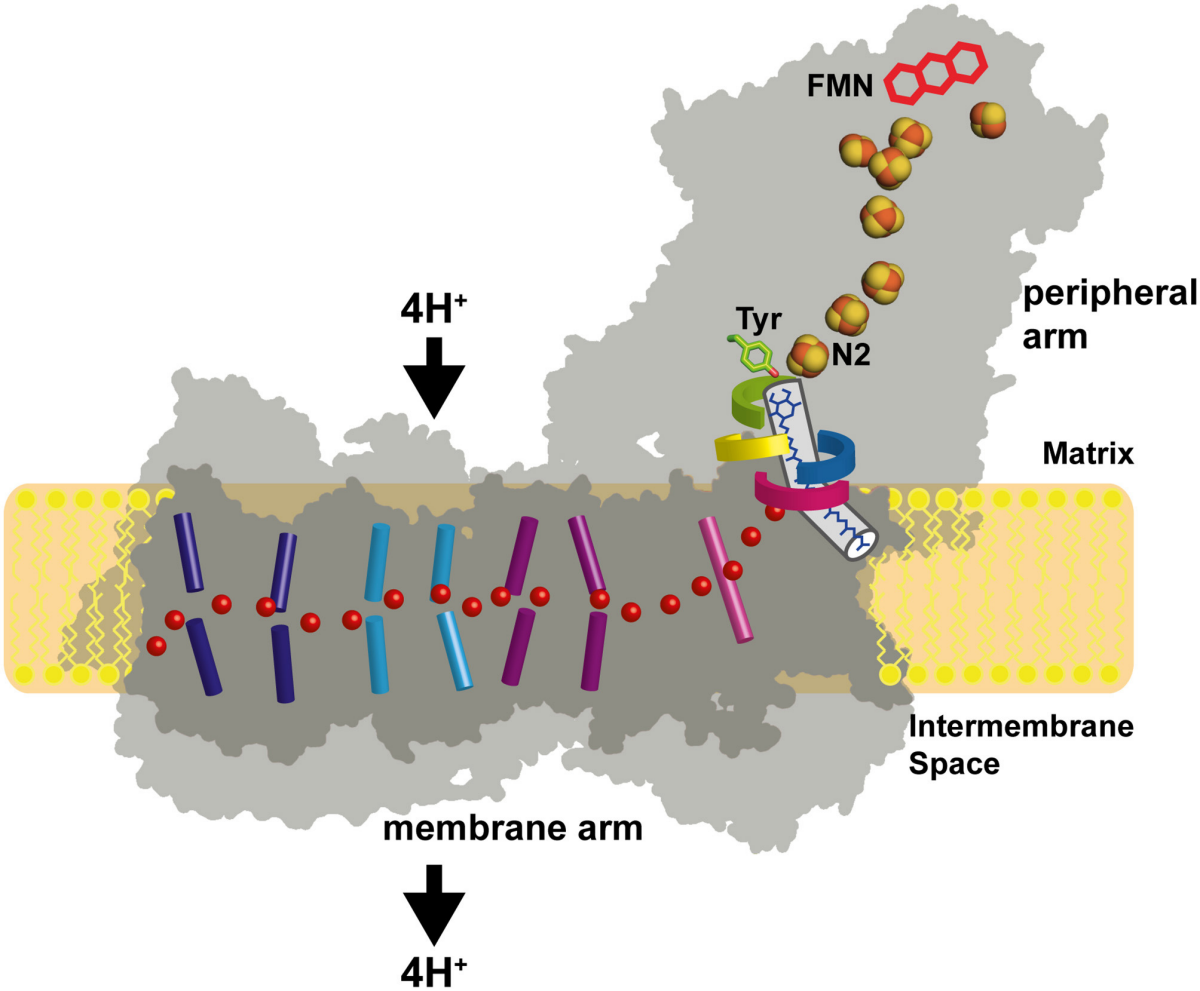
Architecture of complex I. The primary electron acceptor FMN is connected by a chain of FeS clusters with the Q reduction site near FeS cluster N2. Q moves in a tunnel between the active site and the membrane. A hydrophilic axis (red dots) connects the Q module with the proton pumps. Its initial section is called the E channel due to the presence of several strictly conserved glutamates in subunit ND1. Loops of subunits NDUFS2 (green), NDUFS7 (blue), ND1 (red), and ND3 (yellow) line the Q access pathway and the interface between the membrane arm and the peripheral arm. Concerted conformational changes in the loop cluster are thought to play a key role in the coupling mechanism.

Respiratory complex I (also known as NADH dehydrogenase or NDH-1) is a very large membrane protein found in the inner mitochondrial membrane and in the plasma membrane of aerobic bacteria (Hirst, 2013; Sazanov, 2015; Galemou Yoga et al., 2020a). Complex I couples electron transfer from NADH to quinone (Q) to the translocation of protons across the bioenergetic membrane. Note that some bacterial species utilize menaquinone instead of ubiquinone. With a pump stoichiometry of 4 H^+^ per NADH consumed, complex I contributes substantially to the proton motive force that drives ATP synthase. A large variety of compounds are known to inhibit complex I activity by interfering with Q reduction (Murai and Miyoshi, 2016). The catalytic reaction of complex I is fully reversible. In the presence of a reduced Q pool and a sufficiently high membrane potential, complex I can reduce NAD^+^ by reverse electron transfer (RET), e.g., during reperfusion after ischemia (Chouchani et al., 2014). Mitochondrial complex I from many species can switch reversibly from an active A-form to a deactive D-form (Kotlyar and Vinogradov, 1990). The A/D transition is thought to limit the release of detrimental oxygen species under conditions that promote RET (Drose et al., 2016). Complex I dysfunction is associated with neuromuscular and neurodegenerative diseases (Rodenburg, 2016; Fiedorczuk and Sazanov, 2018). The structure of complex I has been determined by x-ray crystallography (Baradaran et al., 2013; Zickermann et al., 2015) and by electron cryo-microscopy (cryo-EM) (Fiedorczuk et al., 2016; Zhu et al., 2016; Agip et al., 2019; Parey et al., 2019, 2020; Grba and Hirst, 2020; Kampjut and Sazanov, 2020; Soufari et al., 2020; Klusch et al., 2021). Cryo-EM structures of the related NADH dehydrogenase-like (ndh) complex or “photosynthetic complex I” have been reported recently (Laughlin et al., 2019; Schuller et al., 2019; Pan et al., 2020). The L-shaped architecture of complex I is highly conserved and consists of a peripheral arm (PA) and a membrane arm (MA) (Figure 1). Fourteen complex I subunits are conserved from bacteria to human. These so-called central subunits harbor all bioenergetic core functions of the enzyme complex. Eukaryotic complex I is much larger than its bacterial counterpart and comprises some 30 additional accessory subunits. The central subunits can be divided into three functional modules. The NADH oxidation module (N module) and the ubiquinone reduction module (Q module) constitute the PA, whereas the proton pumping module (P module) forms the MA of the enzyme. Eight to nine FeS clusters are found in the PA depending on the species. Seven of them connect the primary electron acceptor FMN to the Q reduction site, which is formed by the NDUFS2 and NDUFS7 subunits. Five FeS clusters typically give rise to electron paramagnetic resonance (EPR) signals, namely N1b, N2, N3, N4, and N5 (Ohnishi, 1998; Hirst and Roessler, 2016). Cluster N2 is the last cluster of the electron transfer chain in the PA and the immediate electron donor for Q. The membrane arm of complex I consists of seven central subunits. The three largest subunits ND2, ND4, and ND5 are related to each other and to subunits of bacterial Mrp type sodium proton antiporters (Mathiesen and Högerhäll, 2002). A hydrophilic axis (Baradaran et al., 2013) of titratable residues extending from subunit ND1 at the PA/MA interface to subunit ND5 at the distal end of the MA is thought to play a key role in energy transmission from the Q module to the pump sites. Its connection with the Q module is also called the E channel due to the presence of strictly conserved glutamate residues in ND1. We have proposed that a concerted rearrangement of loops in subunits NDUFS2, ND1, and ND3 is critical for converting the energy released during Q reduction into pump strokes (Zickermann et al., 2015). Indeed, there is now increasing experimental evidence for conformational changes in the Q module and at the PA/MA interface (Agip et al., 2018; Cabrera-Orefice et al., 2018; Galemou Yoga et al., 2019; Grba and Hirst, 2020; Gutierrez-Fernandez et al., 2020; Kampjut and Sazanov, 2020).

In this review, we focus on the progress in the understanding of Q binding and reduction by complex I and its mechanistic implications.

## The Q Reduction Site and the Access Pathway for the Substrate From the Membrane

**Figure 2:**
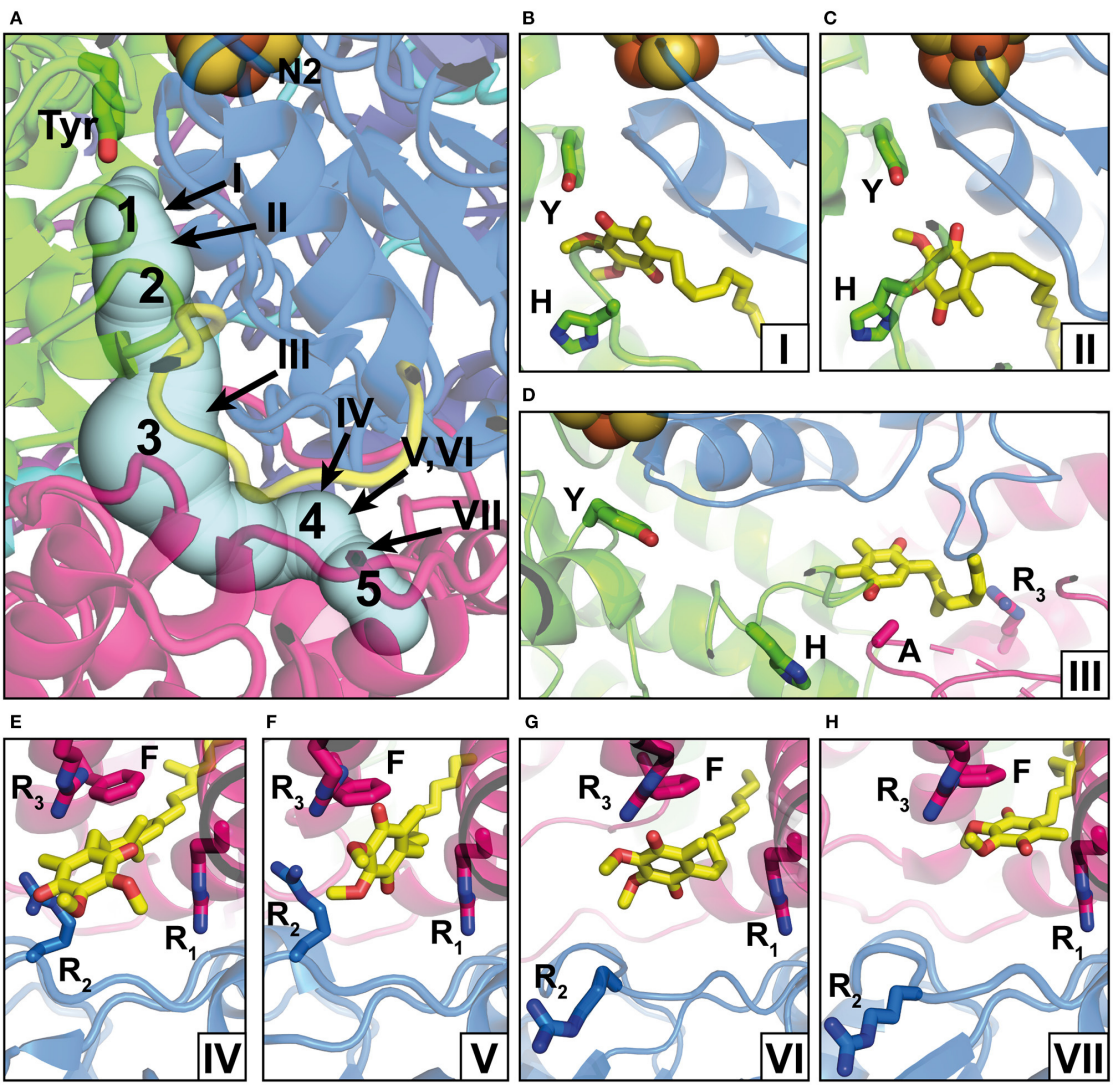
Q binding positions. **(A)** The Q reduction site in the peripheral arm of complex I (PDB: 6RFR) is formed by subunits NDUFS2 (green) and NDUFS7 (blue). A tunnel for Q access from the membrane traverses subunit ND1 (pink). The tunnel was calculated using the CAVER3 software (Chovancova et al., 2012) (starting point conserved Y144, PDB: 6RFR, probe radius 1.3 Å). Intermediate Q binding positions determined by computational methods are indicated by Arabic numbers according to Haapanen et al. (2019). The positions of Q molecules (head group) modeled into X-ray or cryo-EM structures are indicated by Roman numbers and are shown in detailed views in separate panels; the direction of view is consistent for panels in the same row. **(B)** DBQ bound to complex I from *T. thermophilus* (PDB: 6I0D) (Y, Y87; H, H38); **(C)** DBQ bound to complex I from *Ovis aries* in the closed state during turnover (PDB: 6ZKC) (Y, Y108; H, H59); **(D)** PL9 bound to ndh complex I from *T. elongatus BP-1* (PDB: 6KHJ) (Y, Y72; H, H23; A, A237; R_3_, R329); **(E)** Q9 bound to complex I from *Y. lipolytica* (PDB: 6RFR) (R_1_, R27; R_2_, R108, R_3_, R297; F, F228); **(F)** DBQ bound to complex I from *O. aries* in the closed state during turnover (PDB: 6ZKC) (R_1_, R25; R_2_, R77, R_3_, R274; F, F224); **(G)** DBQ bound to complex I from *O. aries* in the open state with NADH bound in the N module (PDB 6ZKH) (R_1_, R25; R_2_, R77, R_3_, R274; F, F224); **(H)** DBQ bound to complex I from *O. aries* in the open state during turnover (PDB 6ZKD) (R_1_, R25; R_2_, R77, R_3_, R274; F, F224).

The position of the Q reduction site in complex I is unique among energy-converting Q-reactive enzymes because cluster N2, the immediate electron donor for Q, resides at around 30 Å above the membrane surface (Figures 1, 2A) (Zickermann et al., 2003). Site-directed mutagenesis studies identified critical residues for Q and inhibitor binding in subunits NDUFS2 and NDUFS7 of the PA (Fendel et al., 2008; Tocilescu et al., 2010b; Sinha et al., 2015). A strictly conserved tyrosine of NDUFS2 was identified to bind the Q head group (Tocilescu et al., 2010a). The x-ray structure of complex I from *Thermus thermophilus* provided structural evidence for the coordination of Q by this tyrosine and by a histidine residue in the loop connecting the first and the second strand of the N-terminal β-sheet of NDUFS2 (Figure 2B) (Baradaran et al., 2013; Gutierrez-Fernandez et al., 2020). This Q binding site is connected by a ~35 Å long tunnel with the membrane bilayer (Baradaran et al., 2013). Site-directed mutagenesis of several residues in the Q access pathway drastically impaired Q reductase activity (Angerer et al., 2012). The entry portal of the Q tunnel is formed by transmembrane helices (TMHs) 1 and 6 and surface helix α1 of subunit ND1. Exchange of an alanine residue in helix α1 interferes with Q reduction kinetics (Zickermann et al., 1998) and is one of the most prevalent causes for Leber's hereditary optic neuropathy (Howell et al., 1991). It has recently been suggested that the entry to the tunnel is so narrow that a conformational change is required to enable the passage of a Q molecule (Wang et al., 2021). The middle of the tunnel is characterized by a highly charged region formed by residues of the TMH5-6 loop of subunit ND1 and a long loop of NDUFS7. Recently, site-directed mutagenesis combined with molecular dynamics simulations identified the critical role of the NDUFS7 loop for binding and the dynamics of Q in the tunnel (Galemou Yoga et al., 2019). Fedor et al. (2017) investigated the impact of the isoprenoid chain length on the kinetics of Q reduction and showed that in contrast to short-chain Q analogs, the dissociation of the long-chain Q10 is not rate-limiting. Movement in the narrow tunnel is thought to be guided by the ~50-Å long isoprenoid chain of Q10 that still reaches into the membrane bilayer when the Q head group is bound at its reduction site near cluster N2. The dynamics of Q in the tunnel have been further studied in three computational approaches (Warnau et al., 2018; Haapanen et al., 2019; Hoias Teixeira and Menegon Arantes, 2019). Free energy profiles consistently suggested the presence of up to five different transient Q binding sites along the Q tunnel. We here follow the nomenclature for intermediate binding sites introduced by Haapanen and Sharma (denoted by Arabic numbers in Figure 2) (Haapanen et al., 2019). Interestingly, these sites largely match with Q binding und in complex I structures determined under different conditions (denoted by Roman numbers in Figure 2). Site 1 is close to cluster N2 at the deepest end of the Q tunnel. Hydrogen bonding of the Q head group with the conserved tyrosine residue (Figure 2B) was reported for crystal structures of complex I from *T. thermophilus*, which was soaked with the short-chain Q analog decyl benzoquinone (DBQ) (Baradaran et al., 2013; Gutierrez-Fernandez et al., 2020). A distance of around 5 Å between the Q head group and tyrosine was recently observed in ovine complex I (Figure 2C) (Kampjut and Sazanov, 2020). This position is similar to the Q binding site identified in the cryo-EM structure of complex I from *Yarrowia lipolytica* captured under turnover (Parey et al., 2018). Although the Q head group has moved away from the tyrosine, this binding position is still assigned to site 1. The two different binding modes might reflect the reduction of Q (Kampjut and Sazanov, 2020) and/or different functional states of the site (see below) (Parey et al., 2018). Site 3 is located approximately in the middle of the Q tunnel and shows some correlation with a bound plastoquinone (PL9) modeled in the recent cryo-EM structure of photosynthetic complex I from *Thermosynechococcus elongatus* (Figure 2D) (Pan et al., 2020). Site 4 is situated in the charged region in the kink of the tunnel at the PA/MA interface. A native Q9 molecule has been observed at this position in complex I from *Y. lipolytica* purified in the detergent lauryl maltose neopentylglycol (LMNG) (Figure 2E) (Parey et al., 2019). Native Q molecules were found in a similar position in plant complex I (Soufari et al., 2020; Klusch et al., 2021). In contrast, a detergent molecule was modeled at this position in complex I from *Y. lipolytica* purified in dodecyl maltoside (DDM) (Grba and Hirst, 2020). Obviously, the more bulky LMNG is unable to enter the narrow opening of the Q tunnel. In the closed state of ovine complex I during turnover, a second DBQ molecule was modeled close to site 4 (Figure 2F). Note that the simultaneous presence of Q molecules in site 2 and site 4 seems only possible because DBQ was used as a substrate. Steric clashes between two Q10 molecules would render a comparable scenario highly unlikely under physiological conditions (Kampjut and Sazanov, 2020). In the open state of ovine complex I with NADH bound, a DBQ molecule was also observed in site 4. It is interesting to note the conformational change of the NDUFS7 loop in this structure (residue R_2_ in Figure 2G). A rearrangement of this loop connected with Q dynamics has been proposed previously (Galemou Yoga et al., 2019). Site 5 is closer to the Q tunnel entrance and binding of Q in this site was observed in the open conformation of ovine complex I during turnover (Figure 2H) (Kampjut and Sazanov, 2020).

Taken together, the evidence for a single narrow Q access pathway in complex I seems to be compelling. However, it should be noted that Uno et al. (2020) showed inhibitor-sensitive reduction of Q analogs which are too bulky to enter the Q tunnel. It is currently unclear how these results can be reconciled with the structural data and more work is needed to resolve this issue.

## Inhibitor Binding Sites in Complex I

**Figure 3:**
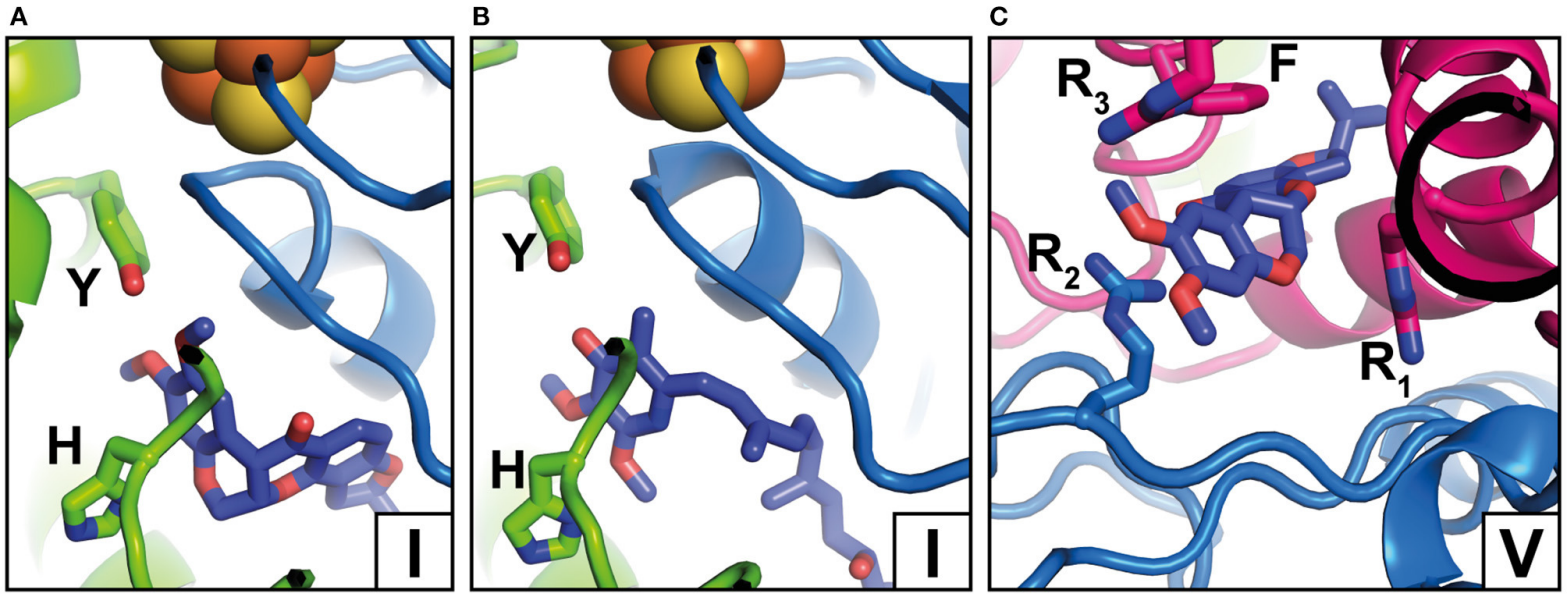
Inhibitor binding positions. **(A)** Rotenone bound to complex I from *O. aries* in the closed state (PDB 6ZKK) (Y, Y108; H, H59); **(B)** Piericidin A bound to complex I from *Mus musculus* (PDB 6ZTQ) (Y, Y108; H, H59); **(C)** Rotenone bound to complex I from *O. aries* in the open state (PDB 6ZKL) (R_1_, R25; R_2_, R77, R_3_, R274; F, F224). Binding positions in the Q tunnel are indicated by Roman numbers and correspond to Figure 2A but note that rotenone occupies a larger area. For colors, see the legend of Figure 2.

Complex I is known to be sensitive to a variety of inhibitors such as piericidins, rotenoids, or quinazolines (Degli Esposti, 1998; Murai and Miyoshi, 2016). Inhibitor binding to complex I was characterized by Scatchard analysis (Gutman et al., 1970), fluorescence quench titrations (Okun et al., 1999), mutagenesis (Darrouzet et al., 1998; Fendel et al., 2008; Tocilescu et al., 2010b; Sinha et al., 2015), and chemical biology approaches (Murai and Miyoshi, 2016; Uno et al., 2018). In recent years, an increasing number of complex I structures with bound inhibitors have become available (Figure 3) (Baradaran et al., 2013; Zickermann et al., 2015; Bridges et al., 2020; Gutierrez-Fernandez et al., 2020; Kampjut and Sazanov, 2020). The binding of three different inhibitors to complex I from *T. thermophilus* was recently analyzed by the Sazanov group (Gutierrez-Fernandez et al., 2020). The crystal structure of the enzyme with bound aureothin, pyridaben, and piericidin A revealed that these inhibitors bind at site 1 at the deepest end of the Q tunnel with their head groups interacting with the essential tyrosine residue near cluster N2. In the crystal structure of complex I from *Y. lipolytica*, the Q antagonist inhibitor 2-decyl-4-quinazolinyl amine (DQA) was modeled near the β1β2 loop of the 49 kDa subunit (Zickermann et al., 2015). More recently, the Hirst group determined the cryo-EM structure of mouse complex I with bound piericidin A (Bridges et al., 2020). Interestingly, two piericidin molecules were found in the Q tunnel. The first inhibitor molecule was bound in site 1 (Figure 3B) in agreement with the position observed in complex I from *T. thermophilus*, while the second molecule was observed at site 4 (not shown). This suggests that the binding of piericidin is cooperative and that piericidin competes with Q for two different binding sites in the Q tunnel. Since rotenone is much bulkier than other Q site inhibitors such as DQA or Piericidin, it has been hypothesized that it cannot enter and transit the narrow Q tunnel. However, in ovine complex I, two rotenone molecules were modeled in the Q tunnel at sites 1 (Figure 3A) and 4 (Figure 3C), respectively. Surprisingly, a third rotenone molecule was found in the ND4 subunit. Note that rotenone has been shown to inhibit Na^+^/H^+^ antiporter activity of deactive complex I (Roberts and Hirst, 2012). The binding of rotenone in the ND4 site could explain this observation. Rotenone binding in the Q tunnel suggests either that the entrance of the Q tunnel undergoes a reorganization to allow access of the bulky molecule or that rotenone can access the Q site *via* alternative pathways (Uno et al., 2018).

## Q Reduction and Mechanistic Implications

It is generally accepted that the energy for driving the proton pumps is released in the Q module and there is increasing evidence showing that the concerted rearrangement of a cluster of four loops surrounding the Q binding site and at the interface of PA and MA (Figure 1) is a central element of the coupling mechanism (Zickermann et al., 2015; Cabrera-Orefice et al., 2018; Parey et al., 2018; Galemou Yoga et al., 2019, 2020b; Kampjut and Sazanov, 2020). Full reduction of Q requires the uptake of two electrons and two protons. The delivery of electrons by a single electron donor and the observation of semiquinone radicals by EPR spectroscopy (Magnitsky et al., 2002) fostered the idea that reaction intermediates accumulate in a stepwise reaction sequence from Q to QH_2_. Early on, the formation of negatively charged quinone species has been proposed to be a key step in the catalytic cycle (Euro et al., 2008). However, note that more recently the assignment of semiquinone EPR signals to complex I has been questioned (Wright et al., 2020). In any case, the timing of charge movements and charge compensation reactions in the Q reduction site is thought to be of utmost importance for the coupling mechanism. Over the years, a number of mechanistic schemes for redox-linked proton translocation have been proposed and a selection of recent models is discussed below.

The stabilization of negatively charged intermediates of Q redox chemistry (Q^.−^ and QH^−^) plays a central role in the two-state stabilization-change mechanism proposed by Brandt (Brandt, 2011). This mechanism assumes two different functional states of the Q binding site. Electron transfer from cluster N2 to Q or QH^.^ is only possible in the so-called E state. Proton transfer to Q^.−^ or QH^−^ is only possible in the so-called P state. Stabilization of the anionic species generated in the E state provides the energy for proton pumping and is tightly linked with transition from the E to the P state. Full reduction of Q to QH_2_ includes two E-to-P state transformations. This does not necessarily mean the execution of two separate pump events because transient storage of electrostatic or conformational energy would still allow pumping in a single step. The cycling between the E and P states is thought to involve a conformational rearrangement of the Q reduction site (Zickermann et al., 2015; Cabrera-Orefice et al., 2018). In fact, we have observed a different mode of Q binding in the cryo-EM structure of complex I from *Y. lipolytica* captured during steady-state activity as compared with Q binding observed in native complex I from *T. thermophilus* (Parey et al., 2018). Different conformations of the β1β2 loop of NDUFS2 in both structures further support the idea of a two-state mechanism associated with concerted loop rearrangements. Recent structures of ovine (Kampjut and Sazanov, 2020) and *T. thermophilus* (Gutierrez-Fernandez et al., 2020) confirm a different mode of Q binding to site 1 (Figures 2B,C). Nevertheless, more work is needed to establish an unequivocal link between the hypothetical E and P states with the protein structure.

Based on structural information with increasing resolution, molecular modeling and molecular dynamics simulation approaches have been established as powerful tools to study complex I function (Hummer and Wikström, 2016). In initial quantum mechanics/molecular mechanics (QM/MM) simulations of Q reduction, Sharma et al. (2015) placed a Q molecule into the Q reduction site and tested the impact of different Q redox states. In case simulations were performed with a Q^2−^, i.e., assuming a two-electron reduction, fast proton transfer from the coordinating tyrosine and histidine residues was observed resulting in the formation of QH_2_. Prior to the proton transfer reaction, the histidine residue forms a salt bridge with a conserved aspartate residue of NDUFS2 that is located further toward subunit ND1. Breaking this ion pair by the redox-coupled proton transfer to Q triggers a conformational change of the β1β2 loop and subsequent flipping of the aspartate side chain is associated with rearrangements of conserved acidic residues in the ND1 subunit. Electrostatic pKa calculations suggested that these changes result in proton uptake from the N side and are thus thought to trigger the loading of the proton pump. Note that the initial formation of Q^2−^ is an essential prerequisite for this mechanism because no proton transfer reactions were observed when oxidized Q or semiquinone states were modeled in the site.

In a later study, Gamiz-Hernandez et al. (2017) reported that the negative charge of cluster N2 shifts the midpoint potential of ubiquinone to a value in the range of −300 mV. This value is unusually low but is in agreement with the values reported earlier based on freeze-quench reduction kinetics (Verkhovskaya et al., 2008) and electrometric calculations (Verkhovskaya and Wikström, 2014). Remarkably, such a dramatic shift in potential would result in an annihilation of the redox potential difference between NADH and a Q molecule in site 1 and would consequently render Q reduction isoenergetic (Wikström et al., 2015; Gamiz-Hernandez et al., 2017; Kaila, 2018). Since, in this scenario, there is a redox potential difference between Q in site 1 and Q in the membrane (+90 mV), the release in free energy is thought to be associated with the movement of Q between site 1 and the exit of the Q tunnel (Wikström et al., 2015; Kaila, 2018). The binding of QH_2_ close to the entry of the E channel, corresponding approximately to site 4, is suggested to “push” out protons previously loaded on acidic ND1 residues by the mechanism described above (Kaila, 2018; Mühlbauer et al., 2020). However, the molecular details of that energy conversion step remain obscure.

The association of complex I with a tightly bound Q molecule was first reported by Verkhovsky et al. (2012) for the enzyme from *Escherichia coli*. Later, native Q molecules were observed in the cryo-EM structures of complex I from *Y. lipolytica* (Parey et al., 2019) and *Brassica oleracea* (Soufari et al., 2020) and free energy calculations suggested that a large energy barrier restricts the movement of Q from the tunnel into the membrane bilayer (Warnau et al., 2018; Haapanen et al., 2019; Hoias Teixeira and Menegon Arantes, 2019). Wikström et al. (2015) have discussed the function of a Q molecule trapped in the Q tunnel to shuttle electrons between cluster N2 and a substrate Q molecule of the membrane Q pool. Haapanen et al. (2019) proposed that the two-electron reduction of a “shuttling Q” by FeS cluster N2 and proton transfer from the nearby tyrosine leads to the formation of a QH^−^ molecule and tyrosinate in site 1. The repulsion of negative charges is thought to drive the movement of QH^−^ to site 4. A substrate Q molecule in site 5 is reduced by electron transfer from the shuttling Q, while the proton released in site 4 is suggested to enter the E channel and to push out protons loaded on antiporter-like subunits. A shuttling Q is known to operate in photosystem II and in bacterial photoreaction centers (Müh et al., 2012). However, for complex I, unambiguous experimental evidence for a comparable mechanistic concept, e.g., the observation of a spin-spin coupled state between two SQ species by EPR spectroscopy, is still lacking.

The recent high-resolution structure of the ovine complex I reported by Kampjut and Sazanov (Kampjut and Sazanov, 2020) has offered a detailed view on Q binding and conformational changes in loops in the Q module and in ND1. Q reduction is thought to involve proton transfer from the Q coordinating histidine and tyrosine residues but in contrast to any previously proposed models, the authors of this study hypothesize that for the re-protonation of site 1, the protons are extracted from two acidic residues in membrane-intrinsic subunit ND4L. The resulting negative charge in this subunit is suggested to subsequently trigger a series of events in the hydrophilic axis that ultimately lead to proton translocation to the P side. However, proton transfer from the membrane interior to site 1 is at variance with two recent studies, which identified putative proton access pathways from the N side (Galemou Yoga et al., 2020b; Grba and Hirst, 2020).

Taken together, the recent surge in high-resolution structural information in combination with molecular simulations and functional studies has greatly advanced the general understanding of complex I. However, conceptually different mechanisms for redox-linked proton translocation are currently discussed. Cryo-EM techniques including tomography as well as computational approaches will become even more powerful in the future. The identification of further intermediates is an avenue for research to comprehensively understand the catalytic cycle of respiratory complex I.

## Author Contributions

EG, JS, and VZ wrote the paper. All authors contributed to the article and approved the submitted version.

## Conflict of Interest

The authors declare that the research was conducted in the absence of any commercial or financial relationships that could be construed as a potential conflict of interest.
